# Exosomes: A new option for osteoporosis treatment

**DOI:** 10.1097/MD.0000000000032402

**Published:** 2022-12-30

**Authors:** Guijiang Huang, Qianhao Zhao, Wenhu Li, Jianlin Jiao, Xin Zhao, Dan Feng, Wei Tang

**Affiliations:** a The First Affiliated Hospital of Kunming Medical University, Kunming City, China; b Kunming Children’s Hospital, Kunming City, China; c Kunming Medical University, Kunming City, China.

**Keywords:** bone remodeling, exosomes, osteoblasts, osteoclasts, osteoporosis treatment

## Abstract

Osteoporosis is a systemic bone disease characterized by reduced bone mass and destruction of bone microarchitecture, leading to increased bone fragility and susceptibility to fracture. However, the pathogenesis and molecular mechanisms of this disease remain unclear. Extracellular vesicles, structures originating from the plasma membrane and ranging from 30 nm to 5 µm in diameter, play an important role in intercellular communication in the bone microenvironment. Exosomes are extracellular vesicles that deliver cargo molecules, including endogenous proteins, lipids and nucleic acids. These cargo molecules are encapsulated in a lipid bilayer and internalized by target cells through receptor-ligand interactions or lipid membrane fusion. With the advancement of exosome research, exosome therapy for osteoporosis is fast becoming a research hotspot for researchers. This review aims to discuss the role of exosomes in the pathogenesis of osteoporosis. In addition, emerging diagnostic and therapeutic properties of exosomes are described to highlight the potential role of exosomes in osteoporosis.

## 1. Introduction

Osteoporosis is a common chronic clinical disease. In the body, osteoclasts and osteoblasts act on the bone to continuously maintain bone resorption and metabolism. Osteoporosis can be triggered when various causes such as estrogen reduction, inflammation, and drugs act on the bone, resulting in increased bone resorption or impaired bone formation, with a series of adverse consequences. The feasibility of stem cell transplantation for the treatment of osteoporosis has been explored in tissue engineering techniques. Although satisfactory results of cell therapy have been achieved in basic research, there are still many challenges such as immune rejection, cell malignancy, and obstacles to the homing of stem cells.^[1]^ Therefore, there is an urgent need to find a treatment method that targets bone regeneration while minimizing the potential harm caused by drug therapy.

Exosomes play an important role in the exchange of information between cells. As early as 1981 Trams et al found in serum-free medium for rat glioma cells culture that the culture medium contained microvesicles with 5´-nucleotidase activity shed by tumor cells.^[2]^ A few years later other researchers reported secretory vesicles in reticulocytes and that these vesicles (50 nm in diameter) were present within large multivesicular endosomes.^[3]^ The vesicles were purified by centrifugation of the reticulocyte culture supernatant and were called “exosomes.”^[4]^ The particle size of exosomes ranges from 30 to 100 nm, while microvesicles show sizes between 100 nm and 1 μm.^[5,6]^ exosomes have been attracting the attention of researchers in some disease-related fields Since their discovery. Current researches on exosomes are focused on the composition and function of exosomes secreted by different cells. As a new form of cell-to-cell communication, exosomes have been implicated in many physiological and pathological processes such as erythrocyte maturation,^[7]^ immune response^[8]^ and tumor migration.^[9]^ Cells integrate genetic material such as nucleic acids and proteins into nano-vesicles to encapsulate cargoes that are secreted outside the cell and nano-vesicles are protected from degradation by proteases and RNA enzymes in the extracellular fluid. Nano-vesicles are transmitted throughout the body, thereby regulating the physiological activities of target cells over long distances. It is reasonable to assume that exosomes play an important role in the process of osteoporosis repair. Ultimately cell-free therapy is a challenging research hotspot that offers new perspectives to explore the pathogenesis and treatment modalities of osteoporosis and has important implications for the design of therapeutic approaches for osteoporosis. In this review, we highlight the importance of exosomes in the pathogenesis of osteoporosis and emphasize the role that exosomes play in bone repair. Finally, we discuss the possible applications of exosomes in the clinical diagnosis and treatment of osteoporosis.

## 2. Exosome formation

Exosomes are membrane vesicles secreted by cells after the fusion of late multivesicular endosomes with the plasma membrane and selectively recruit intracellular proteins, lipids and nucleic acids, reflecting their endosomal biogenesis and explaining their biological functions.^[10]^ Exosome biogenesis begins with the invagination of the plasma membrane to form primary endocytic vesicles, which subsequently fuse to form the early endosomal compartment. During this process, certain proteins are incorporated into the invaginated membrane. While cytoplasmic components are phagocytosed and enclosed in intraluminal vesicles (ILVs). Late intranuclear bodies containing multiple ILVs are known as multivesicular bodies (MVBs). The luminal vesicles of MVBs may bud from the inner surface of the restricted outer membrane of MVBs.^[11]^ Secretion of exosomes into the extracellular requires three pathways: formation of ILVs, prevention of degradation of MVBs and fusion of MVBs with the cell surface. When MVBs fuse with lysosomes, their cargo, including ILV, is degraded. Late intranuclear bodies and MVBs can also fuse with the plasma membrane, releasing their ILV into the extracellular space as exosomes.^[12]^

Exosome formation is a biological process regulated by multiple signaling pathways. Although the exact mechanisms by which exosomes occur remain to be elucidated, some of the cellular components involved in exosome formation and secretion have been identified. The endosomal sorting complex required for transport (ESCRT) complex is the most understood pathway in exosome biogenesis and cargo loading. ESCRT consists of more than 20 proteins that recruit ubiquitinated proteins and other substances that facilitate membrane budding and membrane division into the MVE lumen to form intraluminal vesicles. They are arranged in four protein complexes, ESCRT-0, -I, -II, and -III, which are accompanied by associated proteins such as VPS4, VTA1 and ALIX.^[13]^ ESCRT-0 (vps27, Hse1) is responsible for the recognition and sorting of ubiquitinated proteins.^[14]^ ESCRT-I (vps23, vps28, vps37) and ESCRT-II (vps22, vps36, vps25) act synergistically to promote the invagination of endosomal membranes into buds.^[15,16]^ ESCRT-III (vps2, vps20, vps24, Snf7) form fibrillar multimers that drive vesicles off the membrane into the lumen of the endosome to form intraluminal vesicles.^[17]^ Studies have demonstrated that leptin mediates the upregulation of Tsg101 (a key component of the endosomal sorting complex required for ESCRT-I) expression in breast cancer cells and the leptin/leptin receptor/Hsp90 axis regulates exosome production in breast cancer cells.^[18]^ The synaptic fusion protein 2 (STX2) regulates Rab8a expression in colorectal cancer cells to promote exosome formation.^[19]^ The autophagy protein ATG12-ATG3 has been shown to interact with the ESCRT-associated protein Alix to promote late nuclear endosomal transport to lysosomes and increase exosome biogenesis.^[20]^ Loss of Parkin function results in reduced endosomal tubulation and membrane binding of vesicular protein sorting 35 (VPS35) and sorting nexin 1, as well as reduced mannose 6 phosphate receptor, which leads to increased secretion of exosomes.^[21]^

The production of ILVs by the ESCRT-independent pathway is mainly regulated by sphingomyelin, quadruple transmembrane protein, apolipoprotein E and ceramide (Cer). ILVs can also be formed by a Cer-dependent mechanism. inhibition of neutral sphingomyelinase blocks the conversion of sphingomyelin to Cer and reduces exosome release from oligodendrocytes, suggesting that Cer promotes inward membrane budding and the formation of ILVs.^[22]^ It has been shown that the activity of DEGS1/Ifc to convert dhCer to Cer drives membrane invagination during exosome biogenesis. Overexpression of Ifc inhibits autophagy to increase exosome secretion, but Ifc is not involved in cargo sorting.^[23]^ Continuous Cer catabolism to produce sphingosine 1-phosphate is required to sort exosomal cargo into ILVs, and sustained activation of Gi-coupled S1P receptors on the MVE is essential for sorting cargo into ILVs designed to release exosomes.^[24]^ The cer-rich membrane structural domain serves not only as a permissive environment to promote ILV formation but also as a platform to facilitate the recruitment of ESCRT components. The LC3 and LC3-coupled mechanisms recruit specific substances packed into LC3-positive exosomes for secretion, a process that does not require the ESCRT complex but relies on neutral sphingomyelinase 2 activity.^[25]^ RAB5A encodes a small GTPase required for plasma membrane fusion and early intranuclear body formation.^[26]^ RAB11A is a marker for circulating endosomes.^[27]^ RAB7A is required for late endosome maturation and exosome secretion.^[28,29]^ The Notch4 signaling pathway directly regulates RAB5A and RAB11A, and inhibits RAB7A expression through the Notch4/HES5/RAB7A axis, thereby inhibiting exosome secretion.^[30]^ Acetylheparinase activates the syndecan-syntenin-ALIX pathway to promote endosomal membrane budding and exosome biogenesis by trimming the acetylheparin sulfate chain on syndecan.^[31]^ Small heat shock protein αB is required for exosome secretion by human retinal small pigment epithelial cells.^[32]^ PVT1 promotes co-localization of YKT6v-SNARE homolog (YKT6) and vesicle-associated membrane protein 3. palmitoylation of YKT6 promotes the fusion of MVB with the plasma membrane to promote exosome secretion by pancreatic cancer cells.^[33]^ Active RAB31 participates in FLOTs of lipid raft microdomains, driving EGFR into MVEs to form ILVs and exosomes.^[34]^ Endoplasmic reticulum stress can promote MVB formation and exosome release in different cellular contexts by inducing the unfolded protein response. It has also been shown that stimulation of cervical cancer cells with the membranomycin tunicamycin leads to endoplasmic reticulum stress and increased exosomes release in the endoplasmic reticulum emergency state, and it has been investigated that endoplasmic reticulum stress-dependent exosome release may be mediated by the IRE1 and/or PERK pathway-mediated.^[35]^

Although the mechanism of exosome secretion has been extensively studied, most studies have focused on whether the target promotes exosome secretion, but few have explored in more depth which part of the exosome secretion mechanism (whether it promotes formation or inhibits degradation) the target acts on. There are also differences in the gene regulation of exosomes in different cells. For example, depletion of ALIX in the Hala cell line did not significantly affect total EV secretion, but depletion of ALIX in dendritic cells resulted in a significant decrease in total EV secretion.^[36]^ The results of this study are summarized below. The mechanisms underlying how exosomes are secreted by cells in the bone microenvironment need to be investigated in more depth.

## 3. Uptake of exosomes

Once exosomes are secreted extracellularly. Exosomes’ interactions with receptor cells and the transfer of protein, lipid and nucleic acid contents to target cells regulate their function. But the mechanisms by which receptor cells take up exosomes have not been fully elucidated. The biological activity of exosomes is regulated by various types of endocytosis such as phagocytosis,^[37]^ macrocytosis,^[38]^ lattice-protein-mediated,^[39]^ vesicular protein (vesicle-dependent endocytosis is internalized) mediated,^[40]^ latticin/small concave protein-independent^[41]^ and lipid raft-mediated^[42]^ endocytosis. Phagocytic cells were reported to internalize exosomes derived from human T-cell leukemia cells more efficiently than non-phagocytic cells, with most exosomes observed to attach to the plasma membrane of non-phagocytic cells, whereas in phagocytic cells, these exosomes were found to enter target cells directly via phagocytosis.^[37]^ Different mechanisms of internalization in the same exosome were also suggested.

Differences in the size and surface composition of exosomes may affect their recognition and capture of target cells. Certain cells can capture exosomes in a targeted manner. Although most studies have reported internalization of exosomes by recipient cells. However this is through a nonspecific process such as macro- or microcellular drinking or a specific receptor-dependent pathway is unclear. CD47, an integrin-related protein that protects cells from phagocytosis, is normally present on the surface of exosomes and increases the circulation time of exosomes in the blood by preventing phagocytosis by macrophages and monocytes.^[43]^ Many other proteins localized on the surface of exosomes and receptor cells are thought to be involved in exosomes uptake including integrins, lectins/proteoglycans, T-cell immunoglobulin and mucin structural domain protein 4 (Tim4), which may also contribute to cellular targeting specificity.^[44–46]^ The endonucleosome is the putative site of exosome content delivery, and membrane fusion in response to acidic pH has been suggested as a possible mechanism.^[47]^ Many studies have not assessed the endpoints of exosome internalization such as content delivery, exosomes degradation or re-secretion, which has largely prevented interpretation of the effects that ultimately result from exosomes-mediated cargo transfer.

Lipid substances, such as cholesterol and phosphatidylserine, may also play a role in membrane fusion. For example, the binding of membrane-linked protein V to phosphatidylserine exposed to the surface of certain exosomes can block fusion between monocyte-derived exosomes and activated platelets.^[48]^ However, the surfaces of many exosomes do not contain phosphatidylserine,^[49]^ which suggests that other molecules are also involved. Some unexpected delivery mechanisms, include the transfer of cargo from direct exosomes to the nucleus via contact of these compartments with endonucleosomes containing internalized exosomes^[50]^ or endoplasmic reticulum.^[51]^ The binding of galectin-5 to the surface of rat reticulocyte exosomes regulates exocytosis uptake by macrophages, and overexpression of galectin-5 inhibits exocytosis uptake by macrophages.^[52]^ Epidermal growth factor (EGF) Overexpression of EGF receptor significantly enhanced exocytosis uptake.^[38]^ Bcl-xL (anti-apoptotic protein) acts as a substrate for exosomal caspase-3 and cleavage of Bcl-xL is required for exosome uptake by recipient cells.^[53]^ Clathrin protein-mediated endocytosis is a major endocytosis in exosome uptake.^[54]^ Syncytin-1 and syncytin-2 are detected on the surface of exosomes and mediate the uptake of exosomes by target cells.^[55]^ It has also been shown that the use of exosomes as drug carriers can enable targeted drug delivery, e.g., by adding anti-tumor drugs to exosomes modified in phosphatidylcholine and injecting them into tumor patients to achieve targeted drug delivery.^[56]^ The selective uptake of exosomes by cells has also been demonstrated. The interaction between the cellular integrin α5β1 and the metalloproteinase ADAM17 exposed on the surface of exosomes negatively regulates CD9 playing an important role in the adhesion and uptake of exosomes by colorectal cancer cells.^[57]^ It has been described that disruption of the VOR complex with itraconazole interferes with the transport of exosomal cargo into the nuclear compartment, thereby inhibiting the metastasis of cancer cells’ morphological transformation and their migratory properties.^[58]^

**Figure 1.**
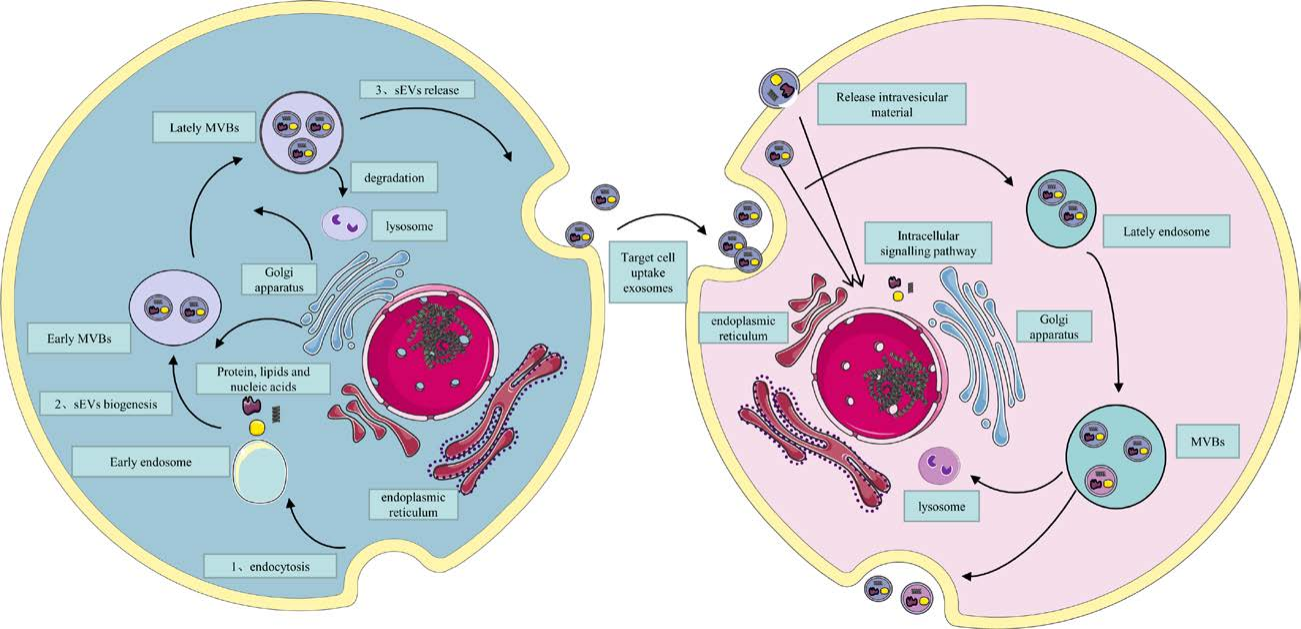
Schematic diagram summarizing of secretion and uptake of exosomes. (1) Secretion of exosomes. the biogenesis of exosomes begins with the invagination of the plasma membrane and the formation of primary endocytic vesicles, which subsequently fuse to form early endocytic luminal vesicles. During this process, certain proteins are incorporated into the invaginated membrane, while cargo is engulfed and enclosed in the luminal vesicles. Endosomes containing multiple endoluminal vesicles are called multivesicular bodies, which may be phagocytosed and degraded by lysosomes or fused to the cytoplasmic membrane, secreting the endoluminal vesicles as exosomes outside the cell. (2) Uptake of exosomes. Exosomes are secreted into the extracellular space and taken up by target cells to mediate cellular responses through different pathways and alter the physiological functions of the target cells. Exosomes can either fuse directly with the plasma membrane or enter the cell and then release their contents thereby generating an intracellular signaling cascade. On the other hand, once the endosome is abandoned by the cell, the contents of the exosome are systematically released into the endocytic compartment and are more likely to be degraded by the fusion of the endosome and lysosome. Some exosomes are also sorted by the luminal vesicles of the target cell and excreted again as exosomes. MVBs = multivesicular bodies, sEVs = small extracellular vesicles.

Almost all the cells we know can produce exosomes, which act as carriers of cellular signaling, transferring nucleic acids, proteins and lipids to target cells, resulting in changes in the phenotype and function of the target cells.^[59]^ Whether and how EV cargo is released and what factors may allow vesicles to enter this specific cell entry pathway will be important questions for future research. The release, formation and uptake of exosomes are shown in Figure 1.

## 4. Exosomes and osteoporosis formation

Exosomes, as messengers of intercellular transmission, are closely associated with the development of osteoporosis. A growing number of studies have confirmed that exosomes contribute to bone loss by signaling to promote osteoclast activity and reduce osteoblast production. High-throughput sequencing comparing the microRNA expression profiles of serum exosomes from menopausal women with osteoporosis and normal women has identified 191 miRNA aberrant expression profiles, with aberrant miRNAs involved in many signaling pathways such as the Wnt, mitogen-activated protein kinase (MAPK) and Hippo pathways.^[60]^ A total of 229 proteins were identified in the proteomics of serum exosomes within the normal, osteopenic and osteoporotic patients. Respectively, 17 proteins were significantly dysregulated in the osteoporotic and osteopenic groups, which involved 10 typical signaling pathways including integrin signaling and actin The actin cytoskeleton signaling pathway.^[61]^ Serum exosomes from 40 normal and 40 osteoporotic patients were also collected and small RNA sequences were performed to detect tRFs (small tRNA-derived fragments) in exosomes. 11 up-regulated tRFs and 18 down-regulated tRFs were identified in osteoporotic patients and the results suggest that plasma exosomes tRF-25, tRF-38, and tRF-18 may be the diagnostic biomarkers for osteoporosis.^[62]^ RNA was extracted from bone marrow mesenchymal stem cells (BMSCs) exosomes from postmenopausal patients and controls, and the differences in CircRNAs between the two groups were analyzed using CircRNA microarrays. 516 differentially expressed CircRNAs were identified, and these differentially expressed CircRNAs were mainly involved in autophagy, PI3K-Akt signaling pathway, FoxO signaling pathway and MAPK signaling pathway and these pathways play important roles in osteogenic differentiation.^[63]^ The number of trap-positive multinucleated osteoclasts in serum exosomes from elderly patients with osteoporosis was significantly higher than the number of osteoclasts in serum exosomes from elderly normal subjects with enhanced bone resorption, but there was no significant difference in the number of trap-positive multinucleated osteoclasts in serum exosomes from elderly normal subjects compared to serum exosomes from young subjects. Osteoblast ALP activity was significantly lower in serum exosomes from osteoporotic patients than in normal human serum exosomes.^[64]^ The expression profiles of microRNA, protein and small RNA sequences of osteoporotic and normal serum-derived exosomes were found to be different by various assays, suggesting a close association between exosomes and the development of osteoporosis in patients with osteoporosis.

In the presence of disease pathology, exosomes secreted by various pathogenic cells can also have an impact on bone formation, with different etiologies resulting in the secretion of exosomes into the circulation for uptake by osteogenic and osteoclast-related cells causing different pathophysiological responses leading to bone loss. It has been demonstrated that in patients with multiple myeloma, serum exosomes are specifically enriched for bimodal proteins and that exosomes can be internalized into mesenchymal stem cells(MSCs), blocking osteoblast differentiation and increasing the release of the osteoclastic factor interleukin-8 to induce osteoclastogenesis.^[65]^ Multiple myeloma cell-derived exosomes can be actively internalized by osteoclast precursor cells to positively regulate osteoclast migration and differentiation.^[66]^ Multiple myeloma exosomes not only enhance osteoclast activity, but also prevent osteoblast differentiation and function in vitro.^[67]^ miR-21 from lung adenocarcinoma cell exosomes promotes osteoclastogenesis by targeting programmed cell death 4.^[68]^ Acute leukemia cell-derived exosomes can alter the bone marrow microenvironment and block bone development and bone formation.^[69]^ Circulating exosomes act as potent regulators of osteoclast formation and blood-derived exosomes from patients with psoriatic arthritis promote osteoclast differentiation.^[70]^ Exosomes from non-small cell lung cancer cells containing AREG induces activation of the EGFR pathway in preosteoclasts, which in turn leads to increased receptor activator of nuclear factor-κB ligand (RANKL) expression and thus induces osteoclastogenesis.^[71]^ miR-26a-5p, miR-27a-3p and miR-30e-5p in exosomes from prostate cancer cells were involved in the inhibition of bone morphogenetic protein 2 (BMP-2)-induced osteogenesis in vivo, and osteogenic progenitor cells exposed to exosomes from prostate cancer cells showed significant downregulation of osteogenic markers and upregulation of proinflammatory factors.^[72]^ The M1 macrophage-derived exosomes co-cultured with osteoblast precursor cells showed a significant upregulation of miR-98 levels in the cells and exacerbated bone loss by downregulating dual-specificity phosphatase 1 and activating the c-Jun N-terminal kinase signaling pathway.^[73]^ In fractured aged females and de-ovulated mice, the osteoclast-derived exosomes miR-214-3p has been transferred to osteoblasts to inhibit osteoblast activity in vitro and reduce bone formation in vivo.^[74]^ miR-139-5p is highly expressed in senescent osteoblasts and their exosomes. miR-139-5p is also upregulated in endothelial cells after treatment of senescent osteoblast-derived exosomes, promoting vascular endothelial cell senescence and apoptosis, and inhibiting endothelial cell proliferation and migration.^[75]^ Levels of microRNA-31a-5p (miR-31a-5p) were significantly increased in exosomes from aged rat BMSCs that increased adipogenic capacity and reduced osteogenesis and cell stemness.^[76]^ Bone marrow macrophage-derived exosomes from type 2 diabetic rats containing miR-144-5P were internalized by BMSCs to prevent fracture healing in rat vivo by inhibiting Smad-1 expression and impeding its osteogenic differentiation.^[77]^ A growing body of research suggests that exosomes play an important role in the development of osteoporosis and that the search for new drugs to target these targets will further benefit osteoporotic patients.

## 5. Exosomes and osteoporosis treatment

**Figure 2.**
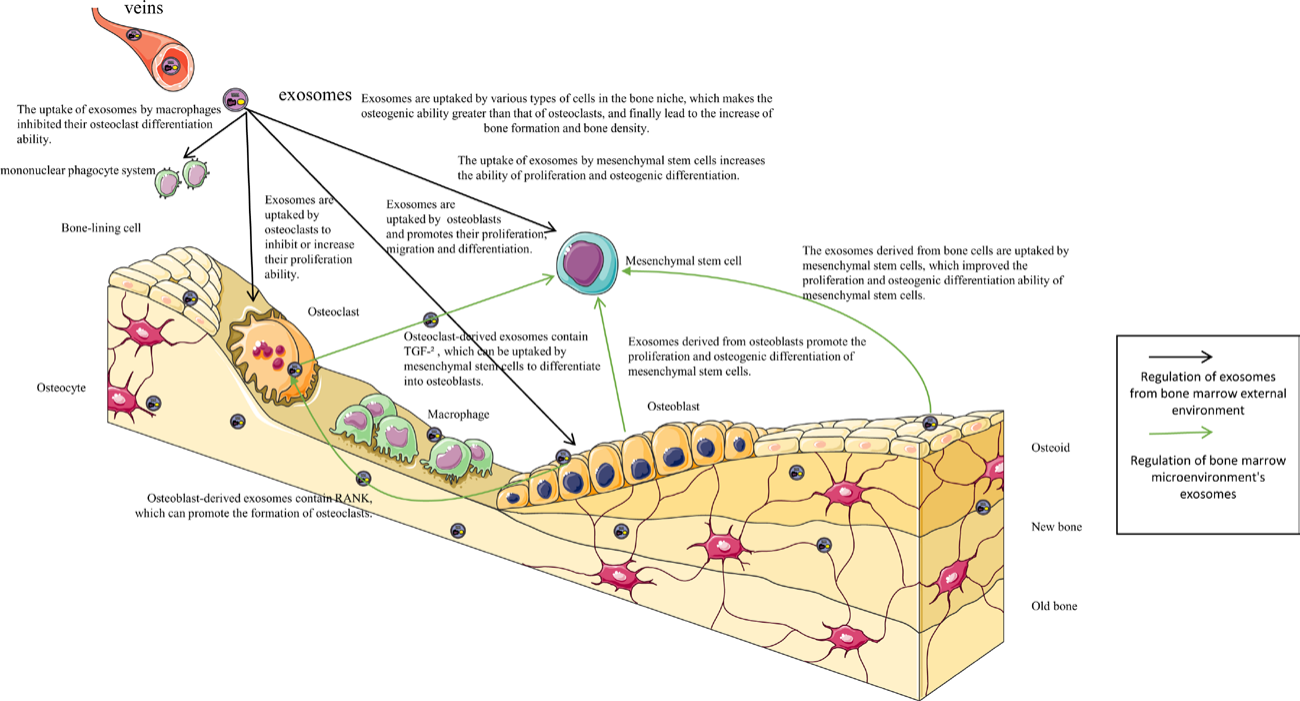
The effect of exosomes on bone remodeling. (1) Exosomes from outside the bone marrow microenvironment reach the bone marrow with the blood vessels and are taken up by MSCs, osteoblasts, and osteoclasts in the bone marrow, regulating the bone marrow microenvironment so that the osteogenic capacity is greater than the osteoclastic capacity, thus increasing bone mass. (2) Exosomes secreted by the bone marrow microenvironment are also involved in the bone remodeling process. For example, osteoclast-derived exosomes act on MSCs to promote the osteogenic differentiation of MSCs, while osteoclast- and osteoblast-derived exosomes can be taken up by MSCs to increase the proliferation and osteogenic differentiation of MSCs. MSCs = mesenchymal stem cells.

Osteoporosis is becoming increasingly common in women after the age of 55 and in men after the age of 65, leading to a plethora of skeletal-related diseases that seriously affect the quality of life of the elderly. The therapeutic effects of conventional drugs are no longer adequate due to poor therapeutic efficacy and significant side effects. Recent studies have shown that exosomes play an important role in skeletal-related intercellular communication. It has been found that exosomes secreted by a variety of cells are involved in the regulation of the proliferation and differentiation of bone-related cells during bone metabolism, and have the advantages of being non-immunogenic and highly targetable. It bridges the deficiency between conventional drugs and stem cell therapy. The schematic diagrams were summarized in Figure 2.

### 5.1. Exosomes and osteoblasts

Osteoblasts are mainly derived from differentiation of MSCs in the periosteum and bone marrow,^[78,79]^ as they play a key role in the synthesis, secretion and mineralization of the bone matrix. Stem cell-based therapy was previously thought to be a new approach to regenerating bone tissue. However, based on several studies, the effectiveness of MSCs in treating osteoporosis is largely dependent on their secretory function rather than their ability to differentiate.^[80]^ It is now generally accepted that exosomes of different cellular origins can be uptake by MSCs and osteoblasts to increase bone formation capacity. Exosomes derived from bone marrow MSCs expressing miR-186 promote YAP expression through regulation of Mob1 and promote osteogenesis through the Hippo signaling pathway in postmenopausal osteoporosis.^[81]^ High expression of miR-1263 in human umbilical cord MSC-derived exosomes effectively inhibits bone marrow MSC apoptosis and prevents osteoporosis in rats by a mechanism mediated by the miR-1263/Mob1/Hippo signaling pathway.^[82]^ Exosomes from young rat bone MSCs were uptake by fatigue-loaded aged osteoporotic rat bone marrow MSCs and that promoted osteogenic differentiation. High expression of miRNA-19b-3p in exosomes improved the osteogenic differentiation of bone marrow MSCs. Overexpression of miRNA-19b-3p targeted a decrease in PTEN expression and significantly increased the expression levels of osteogenic genes such as ALP, type I collagen and runt-related transcription factor 2 (RUNX2).^[83]^ Exosomes from human induced pluripotent stem cell-derived MSCs promoted the proliferation and osteogenic differentiation of osteoporotic rat bone marrow MSCs in vitro and upregulated the expression of osteoblast-associated osteoporotic COL-1, OPN, Runx2 mRNA and protein.^[84]^

Endothelial cell-secreted exosomes antagonize glucocorticoid-induced osteoporosis by inhibiting iron death in osteoblasts.^[85]^ Exosomes from osteoblasts also inversely regulate the differentiation of MSCs, and the uptake of osteoblast exosomes by MSCs increases the expression of Runx2 and Osterix in MSCs, thereby promoting osteogenic differentiation of stem cells.^[86]^ Cui et al showed that exosomes from osteoblast precursor cells interfered with the osteogenic differentiation of bone marrow stromal cells by miRNA in mice, and that the uptake of exosomes from premineralized osteoblasts by bone marrow stromal cells could activate the Wnt signaling pathway by inhibiting Axin1 expression and increasing β-catenin expression, promoting osteogenic differentiation of bone marrow stromal cells.^[87]^ Platelet-rich plasma-derived exosomes promoted the expression of ALP, β-catenin, Runx2 and type I collagen in dexamethasone-treated bone marrow MSCs and preosteoblasts, thereby maintaining osteogenic differentiation and osteogenesis, improving the proliferation of glucocorticoid-treated human microvascular endothelial cells, bone marrow MSCs and osteoblast precursor cells and inhibited cell apoptosis.^[88]^ Exosomes from Bone marrow MSCs carry microRNA-335 that promotes fracture recovery and osteoblast differentiation via the VapB and Wnt/β-catenin pathways.^[89]^ MALAT1-containing exosomes from bone marrow MSCs promote the expression of SATB2 in osteoblasts by mediating the microRNA-34c/SATB2 axis, thereby alleviating osteoporosis.^[90]^ Bone marrow MSC-derived exosomes carrying microRNA-122-5p (miR-122-5p) promote osteoblast proliferation and differentiation by down-regulating the RTK/Ras/MAPK signaling pathway to down-regulate SPRY2 and increasing RTK activity.^[91]^ The exosome miR-181b-5p, secreted by osteoblasts exposed to mechanical strain, was upregulated to promote proliferation of human periodontal stem cells via the miR-181b-5p/PTEN/AKT signaling pathway and osteogenic differentiation of human periodontal stem cells via BMP2/Runx2, suggesting a potential mechanism to maintain periodontal homeostasis.^[92]^ Transforming growth factor β receptor II interacting protein 1 (TRIP-1) is a major intracellular protein in mouse osteoblast precursor cells that can be packaged and exported via exosomes to the extracellular matrix of bone.^[93]^ Upregulation of the AKT signaling pathway in human umbilical cord MSCs-derived exosomes and reduction of osteoclast apoptosis in rat glucocorticoid-induced osteonecrosis via the miR-21-PTEN-AKT signaling pathway.^[94]^ Rat bone marrow MSCs-derived exosomes promote osteoblast proliferation by inhibiting apoptosis and ultimately improving osteoporosis.^[95]^ After co-culture of MSC-EXOs with the osteoblast cell, we found that MSC-EXOs promoted the proliferation of osteoblast and the expression of proteins related to GLUT3 and MAPK signaling pathways in the cells, thereby improving osteoporosis.^[96]^ Myoblast-derived exosomes promoted the differentiation of pro-osteoblasts, and uptake by pro-osteoblasts significantly increased miR-27a-3p levels in recipient cells and decreased APC expression, leading to activation of the β-catenin pathway.^[97]^ Exosomes from Gingival MSCs promote osteoblast migration and osteogenic differentiation of pro-osteoblasts.^[98]^ Inflammatory osteoblast-derived exosomes containing lncRNA LIOCE were uptaked by osteoblast precursor cells, which significantly increased the expression of ALP and Runx2 in osteoblast precursor cells after uptake and reduced their ubiquitination levels by interacting with Osterix, stabilized the osteogenic transcription factor Osterix and promoted bone formation in mice with inflammatory osteolysis.^[99]^ The miR-206 in bone marrow MSC-derived exosomes increased ALP and OCN activity and promoted proliferation and inhibited apoptosis of osteoblasts in a model of osteoarthritis by inhibiting Elf3 expression.^[100]^ Bone marrow MSC-derived exosomes containing miR-150-3p promoted osteoblast proliferation and differentiation by promoting the expression of Runx2 and Osterix in both in vivo and in vitro experiments.^[101]^

Exosomes derived from various tissues and organs in the body can be taken up by osteoblasts or MSCs to influence their physiological functions, suggesting that exosomes play an important role in the differentiation of osteoblasts. This provides new insights into the pathogenesis and treatment of osteoporosis. Based on the physiological functions of these exosomes will potentially open up new pathways for the future development of osteoporosis drugs.

### 5.2. Exosomes and osteoblasts

Bone remodeling is essential for the repair and replacement of damaged old bone, the process is largely mediated by osteoclast-mediated bone resorption followed by the recruitment of preosteoblasts followed by differentiation to produce osteoblasts. Growth factors are known to be released in large quantities during bone resorption by osteoclasts, transforming growth factor-β and insulin-like growth factor-1 are known to stimulate osteoblast differentiation and are activated by the acidic pH produced by osteoclasts during bone resorption.^[102,103]^ It has also been shown that the anabolic action of the parathyroid gland is dependent on the presence of osteoclasts.^[104]^ Osteoclasts stimulate bone formation and thus lead to anabolic effects.^[105]^ Osteoclasts are essential for bone formation and it has been shown that individuals with loss of receptor activator of nuclear factor-κB (RANK)/RANKL signaling not only have disrupted bone resorption, but also have greatly reduced bone formation.^[106]^ There is increasing evidence that osteoclasts direct osteoblasts to form bone and that miR-223 molecules as an important regulator of osteoclastogenesis contained in macrophage-derived microvesicles are transported to macrophages to participate in osteoclast differentiation.^[107]^ Adipose-derived MSCs have great potential to suppress inflammation, and AD-MSCs-derived exosomes inhibited IL-1β and IL-18 secretion in high-glucose-treated osteoclasts and restored streptozotocin-induced bone loss in diabetic osteoporotic rats, and inhibited NLR family, pyrin domain containing 3 inflammasome activation in osteoclasts to reduce diabetic osteoporosis.^[108]^ Colostrum-derived exosomes significantly inhibited the number of macrophage cells stained for acid phosphatase against tartrate indicating reduced osteoclast differentiation, and mice orally administered bovine milk exosomes exhibited significant anti-osteoporotic effects, but the mechanism of entry from the gastrointestinal tract into the body is unclear.^[109]^ It has also been shown that the presence of milk-derived extracellular nanoparticles promotes the differentiation of preosteoclasts into osteoclasts, with an increase in the number of osteoclasts with ≤5 nuclei and an increase in the expression of TRAP, NFATc1, and c-Fos, but no effect on the number of osteoclasts with large nuclei.^[110]^ RANKL was expressed in exosomes secreted by osteoblasts and stromal cells and is transferred to osteoblast precursor cells to promote differentiation.^[111]^ miR-1260b in gingival tissue-derived MSC-derived exosomes targets the Wnt5a-mediated RANKL pathway and inhibits its osteoclast activity.^[112]^ Exosomes from osteoblast precursor cells containing circ_0008542 promotes osteoclast differentiation and bone resorption through m6A methylation and upregulate RANKL gene expression levels.^[113]^ TI-induced purified exosomes from mouse macrophages inhibit osteogenic differentiation of pro-osteoblasts.^[114]^ Vascular endothelium-derived exosomes have more potent bone targeting than osteoblast-derived or bone marrow MSC-derived exosomes, and high expression of miR-155 inhibited osteoclast activity in vitro and suppressed osteoporosis in an ovariectomized mouse model.^[115]^ Osteoclast-derived exosomes can target osteoblast precursor cells and promote their osteogenic differentiation.^[116]^ Moreover, exosomes released from mineralized osteoblasts contain miR-503-3P, which can be targeted by osteoclasts to inhibit osteoclast differentiation by down-regulating the expression of Hpse.^[117]^

### 5.3. Exosomes promote angiogenesis

Blood vessels mediate the transport of oxygen, nutrients and waste products to and from cells. In the human skeletal system, the growth of the vascular network is regulated by signals provided by chondrocytes and other bone cells, and blood vessels play an important role in the formation of bone.^[118,119]^ Vascular endothelial growth factor (VEGF) is a popular area of research. VEGF induces β-linked protein transcriptional activity in endothelial cells and osteoblasts via VEGF receptor 2- and phosphatidylinositol 3-kinase to increase bone mass.^[120]^ The structure of the osteo-vascular system is closely related to the process of bone ecotone regulation. The capillaries of the mouse skeletal system can be divided into H-type and L-type endothelium based on morphology, molecular and function. H-type endothelial cells mediate the local growth of the vascular system and provide ecotone signals to perivascular osteoprogenitor cells, and specific molecular pathways can be used to promote the formation of H-type vessels and osteogenesis.^[118]^ Vascularity in bone is essential for the maintenance of bone homeostasis, and several exosomes have been shown to increase bone density by promoting angiogenesis in the bone ecotone. It has been found that hiPSC-MSC-exosomes stimulate angiogenesis and bone regeneration in vivo and in vitro.^[84]^ Zhang et al found that bone marrow MSCs exosomes significantly increased osteogenesis, angiogenesis and fracture healing in a rat model of femoral dysplasia, were taken up by human umbilical vein endothelial cells and pro-osteoblasts and led to improved value addition and migration, possibly due to activation of the BMP-2/Smad1/RUNX2 and HIF-1α/VEGF signaling pathways.^[121]^ Endothelial progenitor cell-derived exosomes overexpressing lncRNA-MALAT1 bind directly to miR-124 to negatively control miR-124 activity, and their exosomes induce macrophages migration and osteoclasts differentiation in vivo and increase angiogenesis at the fracture site.^[122]^ VEGF, bFGF and PDGF-BB were highly expressed in PRP-exosomes and had a significant protective effect on the femoral vasculature in a rat model of glucocorticoid-induced femoral head necrosis.^[88]^

Other exosomes, although not identified for their effect on bone, have been shown to affect vascular endothelial cells to promote angiogenesis and may be potential targets for the treatment of osteoporosis. It has been shown that MSC-derived exosomes can be taken up by umbilical vein endothelial cells in vivo to promote angiogenesis and protect myocardial tissue from ischemic damage.^[123]^ Adipose MSC-derived exosomes can mediate the angiogenic activity, and exosomes released by adipose-derived stromal cells may contribute to adipose-derived stromal cells-induced angiogenesis, suggesting that PDGF may trigger the release of exosomes with enhanced angiogenic potential.^[124]^ Adipose MSC-derived exosomes can be taken up by human umbilical vein endothelial cells and promote angiogenesis in vitro.^[125]^ Microvesicles released from endothelial progenitor cells promote the vascularization of the human pancreas islet.^[126]^ Exosomes release from placenta derived mesenchymal stem cells is increased by hypoxia and promotes endothelial cell migration and angiogenesis in a concentration and oxygen-dependent manner.^[127]^ MMP-2, which is present in the exosomes of mature osteoblasts, promotes endothelial angiogenesis in vitro via the VEGF/Erk1/2 signaling pathway.^[128]^

### 5.4. Exosome suppression of inflammatory response for osteoporosis

The inflammatory response may lead to a decrease in bone mass and even to the development of osteoporosis in the body, for example in rheumatoid arthritis,^[129]^ Crohn’s disease,^[130]^ systemic lupus erythematosus and psoriatic arthritis.^[131]^ Exosomes appear to play a key role in multiple signaling cascades during inflammation, as they can carry inflammatory regulators such as miRNAs and proteins that can act on both proximal and distal target tissues. Promoting bone repair is a novel target for the use of exosomes in the treatment of inflammatory bone loss, and better outcomes may be achieved when therapeutic strategies that promote bone repair are combined with those that inhibit inflammation. In the measurement of the pro-inflammatory factor IL-6 in the bone marrow supernatant of osteoporotic women, a significant increase in IL-6 levels was found in comparison to the normal population of osteoporotic female patients.^[132]^ Under inflammatory conditions, the release of pro-inflammatory factors such as tumor necrosis factor and IL-6 leads to cellular suppression. Although in many cells the nuclear factor kappa B (NF-κB) pathway is involved in cell development and function, in contrast NF-κB activation inhibits osteoblast activity and inhibition of IKK-NF-κB enhances the expression of Fos-related antigen 1 to increase bone matrix formation.^[133]^ TNF, IL-1, and IL-6 all affect osteoclastogenesis.^[134]^ Bone marrow MSC-derived exosomes express CCR2, which has a high capacity to bind its ligand CCL2, and MSC-exosomes with high CCR2 expression can reduce free CCL2 concentration and inhibit macrophage function.^[135]^ Both MSCs-derived microvesicles and exosomes have anti-inflammatory effects on T and B lymphocytes and have therapeutic potential for bone strength recovery.^[136]^

### 5.5. Modified exosomes for osteoporosis

Exosomes are a newly investigated mechanism of intercellular communication involving the transfer of molecules, and biotechnology now allows for the structural modification and engineering of exosomes.^[137]^ Includes modifications to exosomal chemical linkers, proteins, lipids and nucleic acids.^[138]^ Altering specific substances in exosomes or using tissue engineering techniques to quantitatively densify exosomes can be an effective treatment for osteoporosis. There are many ways to modify exosomes, and many physical modifications have made satisfactory progress in basic research. For example, the use of nano-localized calcium sulphate/nano-hydroxyapatite as a carrier for BMP2, zoledronic acid and bone marrow MSC-derived exosomes significantly enhances bone formation and defect healing and reduces the use of BMP2 in a rat model of osteoporotic femoral neck canal defects.^[139]^ The use of a porous β-TCP scaffold as a carrier for hiPSC-MSC-exosomes to repair cranial defects significantly improved osteogenesis and increased neointimal regeneration in a cranial defect model compared to the scaffold without hiPSC-MSC-exosomes.^[84]^

Additionally loading exogenous proteins or nucleic acids into exosomes is a viable way to explore the use of modified exosomes for disease treatment. Co-culture of bone marrow MSC-transfected circRNARtn4 (circ-Rtn4)-secreted exosomes attenuated TNF-α-induced murine preosteoblast toxicity and apoptosis by sponging miR-146a.^[140]^ Delivery of microRNA-935-modified bone marrow MSC-derived exosomes to osteoblasts inhibited STAT1 levels and enhanced osteoblast proliferation and differentiation in osteoporotic rats.^[141]^ Overexpression of microRNA-5106 in M2 macrophage-derived exosomes, which directly targets the SIK2 and SIK3 genes to induce osteogenic differentiation of bone MSCs.^[142]^ Levels of microRNA-31a-5p (miR-31a-5p) were significantly increased in aged rat MSC exosomes, and inhibition of miR-31a-5p significantly inhibited osteoclast activity and prevented bone loss in aged rats.^[76]^ Overexpression of lncRNA H19 human umbilical cord MSC-derived exosomes upregulated FoxO3 in chondrocytes to effectively prevent cartilage defects.^[143]^ Overexpression of miR-486-5p in exosomes secreted from rheumatoid arthritis fibroblast-like synoviocytes promotes osteoblast differentiation by activating the BMP/Smad signaling pathway and inhibiting Tob1.^[144]^ Modification of cargoes in exosomes has also yielded significant results, and the next step in the study is to apply the findings from the basic experiments to the clinical setting for the benefit of patients. The main challenge in modifying exosomes is to maintain the biological function of the exosome during the modification process. In summary, appropriate modification and engineering of exosomes enhance the therapeutic potential through different aspects, such as enhanced targeting ability or loading of therapeutic agents. However, engineering exosomes without compromising their biological activity and function has been a key issue.

## 6. Clinical research and application of exosomes in patients with osteoporosis

Exosomes can be a powerful potential marker for the diagnosis and prevention of osteoporosis. Different cells secrete different intra-exosomal cargoes and the same cells secrete different exosomal cargoes under different conditions, Pathological changes in the tissue microenvironment are reflected in the exosomal cargoes they release. Cells in pathological states have been shown to increase their rate of exosome release, which has been demonstrated in cancerous diseases.^[145]^ This suggests that they may act as a biomarker in the development of disease. Serum exosomes from healthy people and patients with osteoporosis contain different cargoes that may be measured as biomarkers for the diagnosis and prognosis of osteoporosis. However, there are many difficulties in purifying and stably extracting exosomes. the currently used differential centrifugation method has many disadvantages, different centrifugal forces are used for the optimal separation of exosomes from different cells, for example, the optimal vesicle protein yield is 67,000 g for HEK293 cells and 100,000 g for FL3 cells^[146]^ and there is co-separation of impurities, low reproducibility and potential damage to exosomes. It has also been shown that there are differences in the extraction of the same exosome cargo by different exosomes extraction methods.^[147–149]^ With the continuous development of extraction techniques, ultrafiltration has shown great potential in exosomes isolation techniques for processing and analyzing large amounts of exosomes isolated from human blood and plasma. But yields are highly dependent on the user and the equipment used, in addition to the fact that centrifugation techniques require not only expensive instruments but also time-consuming procedures. The buffered density gradient ultracentrifugation method can produce different layers with different densities and exosomes can be localized at different buoyant densities in the layers, using this method allows better differentiation of exosomes. However, the buffered density gradient ultracentrifugation method requires more time for sample purification.^[149]^ Each current method of exosomes extraction has its advantages and disadvantages, for example recent microfluidic devices have been successfully used to isolate exosomes from different samples including human plasma, serum and urine. However each microfluidic based method has some advantages and disadvantages and no one device can be used effectively for all types of samples.^[150]^ In the future, if exosomes are to be used for the clinical diagnosis and prognosis of osteoporosis, it will be necessary to improve the method of extraction of exosomes to obtain stable exosomes to ensure stable and reproducible experimental results. Exosomes are already being used to diagnose AIDS, Alzheimer’s disease and cancer.^[151,152]^ As technology continues to advance, it has been shown that sensors will become more versatile when integrated with micro-units, plasmonic sensors provide real-time and label-free detection of biological targets with unprecedented sensitivity and detection limits.^[153]^ Plasmonic sensors not only enable the separation of exosomes, but also allow in situ analysis on the sensors.^[154]^

Exosomes of different cellular origins and containing different components have demonstrated bone regeneration, which makes exosomes therapy for osteoporosis possible. Exosomes, as nanoscale vesicles of natural origin, have advantages that cannot be replaced by other synthetic nanomaterials, such as low immunogenicity, non-toxicity and the ability to be endogenized by target cells, making them a hot topic for research. Moreover, the use of exosomes as an alternative to cell therapy may be more direct and safer due to their tiny structure that can participate in the systemic circulation. With advances in biotechnology, exosomes are more easily extracted and modified. In addition, exosomes, as self-biologically active substances, can cross many barriers that drugs cannot, and drug modification of exosomes can increase the therapeutic effect by targeting the uptake of drugs to the site of the lesion. It has been shown that intravenous RVG-targeted exosomes specifically deliver GAPDH siRNA to neurons in the brain.^[155]^ The presence of CXCR4 within mouse embryonic fibroblast-derived exosomes allows for targeted enrichment of exosomes in the bone marrow, we fused CXCR4 exosomes with liposomes carrying antagomir-188 to produce hybrid nanoparticles that acquire bone-targeting capabilities to release antagomir-188 in bone marrow in a targeted manner to promote BMSC osteogenesis and inhibit lipogenesis.^[156]^ Exosomes are used for three main purposes in the treatment of osteoporosis, targeted modulation of the balance between osteoclasts and osteoblasts, structural modification of exosomes and exosomes as drug carriers. Although the identity and function of some of the cargoes contained in exosomes have not been determined, it has been shown that exosomes can promote bone regeneration in animal models. Exosomes with active ingredients can treat a variety of skeletal disorders including osteoporosis and osteoporotic fractures. Several exosome-based drug delivery systems are currently under development for use in disease treatment and are awaiting clinical trials. In addition, there are several other uses for exosomes in disease treatment that require further research.

## 7. Summary and outlook

In summary, naturally occurring exosomes have a variety of active components and play an important role in the treatment of osteoporosis, without causing the immune response that may result from cell therapy due to defects in the expression of MHC-I and MHC-II proteins on the surface of exosomes. And excellent results have been achieved in basic research using structural modifications and vector constructs of exosomes for the treatment of osteoporosis. Although important studies have been carried out on exosomes, many questions remain to be answered regarding the identity, function, and mechanisms of the molecules present in exosomes, their secretion and transport uptake, and many other aspects of exosomes.

Although exosomes have become a research hotspot in the medical field, their practical application in osteoporosis still requires considerable research due to a variety of other factors including their high cost that needs to be addressed.

## Author contributions

**Conceptualization:** Guijiang Huang, Qianhao Zhao, Wenhu Li, Jianlin Jiao, Xin Zhao, Dan Feng, Wei Tang.

**Data curation:** Guijiang Huang.

**Formal analysis:** Guijiang Huang.

**Writing – original draft:** Guijiang Huang, Qianhao Zhao, Wei Tang.

**Writing – review & editing:** Guijiang Huang, Wei Tang.
